# Recurrent strokes as the sole manifestation of antineutrophil cytoplasmic antibody associated vasculitis in a patientwith panuveitis: a case report

**DOI:** 10.1186/s12348-025-00454-0

**Published:** 2025-02-20

**Authors:** Hadeel Seraj, Hanan A. Alshalan, Reham Alemam, Mohammed A. Omair

**Affiliations:** 1 Vitreoretinal Division, King Khaled Eye Specialist Hospital, Riyadh, Saudi Arabia; 2 Rheumatology Unit, Department of Medicine, King Fahad Specialist Hospital, Tabuk, Saudi Arabia; 3 Rheumatology Unit, Department of Medicine, King Saud University, Riyadh, Saudi Arabia

**Keywords:** Antineutrophil cytoplasmic antibody (ANCA)-associated vasculitides (AAV), Panuveitis, Cytoplasmic anti-neutrophil cytoplasmic antibody, c-ANCA, Necrotizing inflammation, Ocular manifestations, Cyclophosphamide

## Abstract

Antineutrophil cytoplasmic antibody (ANCA)-associated vasculitides (AAV) are a heterogeneous group of rare, autoimmune conditions characterized by widespread, multisystemic inflammation of small to medium-sized blood vessels. We present a case report of a 46-year-old male with a history of prior ischemic strokes and recurrent bilateral non-granulomatous panuveitis associated with a strongly positive cytoplasmic anti-neutrophil cytoplasmic antibody (c-ANCA) titer. Initial treatment with steroids, methotrexate, and rituximab were ineffective, but the condition responded moderately to cyclophosphamide. This case underscores the importance of considering AAV in patients with unexplained ocular inflammation and highlights the role of c-ANCA testing in diagnosing and managing such cases, even in the absence of classic systemic symptoms.

## Introduction

Antineutrophil cytoplasmic antibody (ANCA)-associated vasculitis (AAV) are a heterogeneous group of rare, autoimmune conditions characterized by widespread, multisystemic inflammation of small to medium-sized blood vessels [1]. Previous research on AAV has indicated that the prevalence of ocular involvement is around 16% [2]. Granulomatosis with polyangiitis (GPA) is a rare subtype of AAV characterized by life-threatening necrotizing multi-system inflammation. Among AAV, ocular involvement is most frequently associated with GPA [3]. While orbital disease and scleritis are the most common presentations, AAV can also manifest as conjunctivitis, peripheral ulcerative keratitis, uveitis, and retinal vasculitis [4]. Although well described in the literature, ocular manifestations as the “initial” presentation of AAV are uncommon (6%). One study had reported scleritis as the presenting feature of AAV, which mandate comprehensive investigations that establish the diagnosis before the patient develops severe and life-threatening pulmonary haemorrhage [5].

Furthermore, patients with ocular manifestations of AAV had a mortality rate 2.75 times higher than those with other inflammatory eye conditions. Therefore, timely recognition of these ocular signs and their underlying systemic association is crucial for reducing both morbidity and mortality [4].

Herein, we are reporting an unusual case of AAV manifesting with panuveitis as the initial presentation that led to the diagnosis in a patient with a prior history of unexplained recurrent ischemic strokes.

## Case presentation

### History of presenting illness

**Fig. 1:**
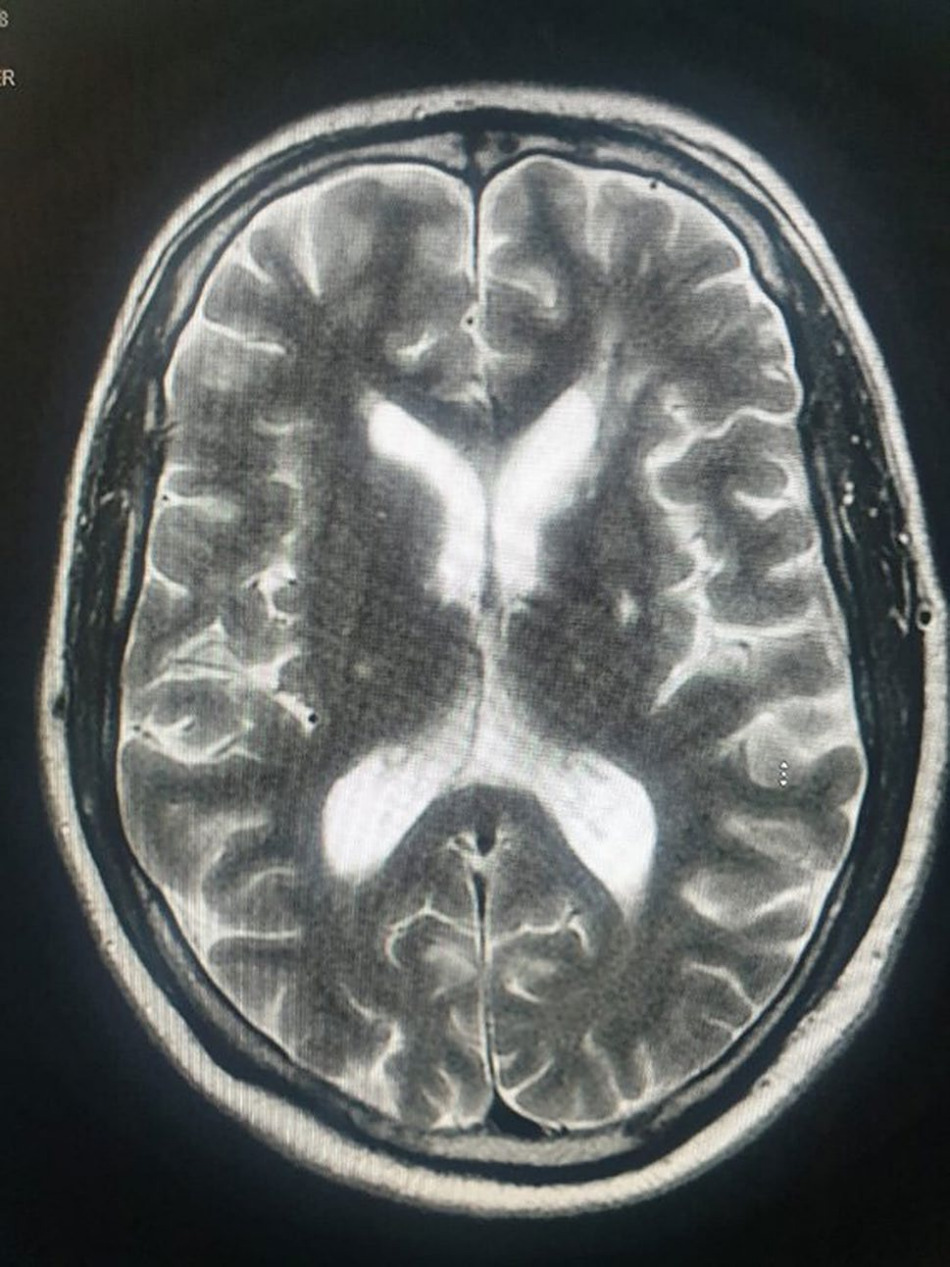
Magnetic resonance imaging utilizing stroke protocol showing small foci of high T2/FLAIR signal intenisty

A 46-year-old male was referred to our department for the evaluation of chronic idiopathic non-granulomatous anterior uveitis that had proven resistant to both topical and systemic prednisolone treatments. The patient reported persistent bilateral eye pain and redness, along with a progressive decline in vision over the past six years. His medical history is notable for moking six packs per day for over 20 years. He was enrolled in a smoking cessation clinic and successfully transitioned to nicotine patches. He had several minor ischemic strokes, which were manifested with dysarthria and limb weakness. Magnetic Resonance Imaging (MRI) revealed nonspecific white matter lesions with T2 hyperintensities (Fig. 1). He was managed with daily aspirin and rivaroxaban. Additionally, he has undergone a splenectomy due to severe autoimmune haemolytic anaemia, which was refractory to corticosteroid treatment and complicated by pneumonia and pleural effusion.

### Ophthalmic examination

**Fig. 2:**
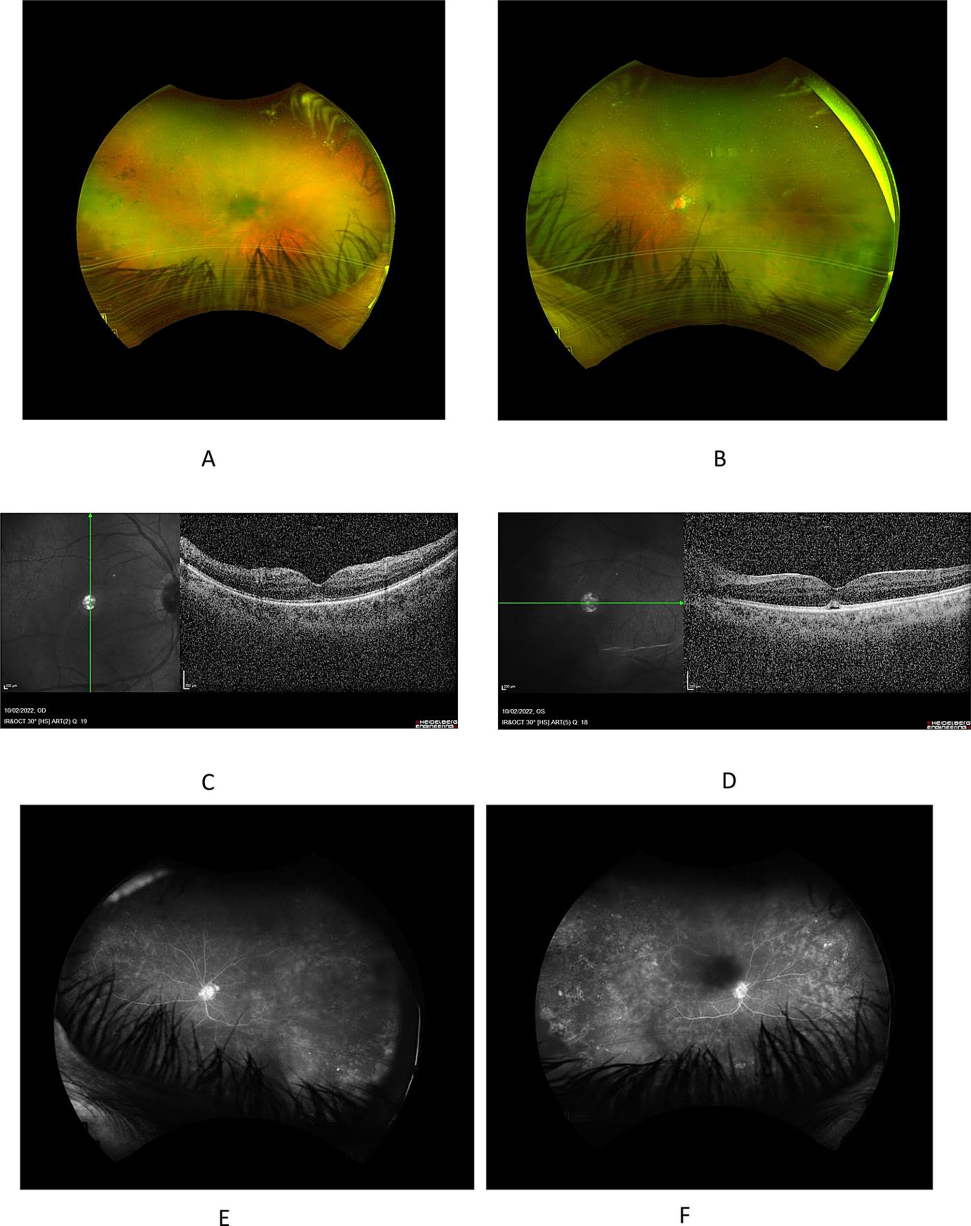
Visit Date: 17/3/2022. Optos fundus photos at presentation. (**A**) Right eye showing vitreous condensation with peripheral pigmented scars, and no clear vasculitis or retinitis. (**B**) Left eye showing vitreous condensation with peripheral pigmented scars, and no clear vasculitis or retinitis. (**C**) Optical coherence tomography (OCT) at presentation of the ight eye showing dry macula. (**D**) Left eye showing dry macula. (**E**) Late frames of fundus fluorescein angiogram at presentation of the right eye revealing temporal peripheral ischemic areas with late diffuse capillary leakage and hot disc. (**F**) Left eye revealing temporal peripheral ischemic areas with late diffuse capillary leakage and hot disc

Ophthalmic examination upon presentation on the 17th of March 2022 revealed visual acuities of 20/100 in the right eye and 20/160 in the left eye and intraocular pressures of 13 and 12 mmHg, respectively. Anterior segment examination showed bilateral conjunctival hyperaemia and clear corneas with no keratic precipitates. Both eyes showed deep anterior chambers with flare and 2 + mixed cells (grading based on the Standardization of Uveitis Nomenclature Working Group criteria [6]), fibrotic pupillary membranes with peripheral anterior synechiae, and posterior subcapsular cataracts corresponding to the chronicity of the condition. These findings resulted in a poor view of the posterior segment. However, upon fundus examination, there appeared to be vitreous haze with condensations, bilateral peripheral pigmented chorioretinal scars, and no clear vasculitis or retinitis (Fig. 2-A, B).

An optical coherence tomography (OCT) was obtained, showing tiny subretinal fluid in both eyes (Fig. 2C, D). Fundus fluorescein angiography (FFA) was also performed, revealing temporal peripheral ischemic areas with late diffuse capillary leakage and a hot disc in both eyes (Fig. 2E, F).

### Diagnosis and uveitis workup

Based on the examination and FFA findings, he was labelled as having chronic bilateral non-granulomatous panuveitis. This required further investigations to assess the possible underlying cause and tailor the treatment accordingly. A thorough laboratory workup for common infectious aetiologies in the Saudi region was carried out. For tuberculosis, he had a prior history of one positive Quantiferon reading (for which he was started with Isoniazid (INH) elsewhere); this was followed by discontinuation of INH due to two negative Quantiferons and three negative purified protein derivatives (PPD) tests. Screening for hepatitis and syphilis came back negative. Given the patient’s history of ischemic strokes, a comprehensive evaluation for other rheumatological causes was conducted. This assessment revealed a strongly positive c-ANCA result, measuring 40 units/ml. (Reference Range: Negative: ≤19 AU/mL, Equivocal: 20–25 AU/mL, Positive: ≥26 AU/mL) with positive anti-PR3 antibodies.

Computed tomography of the chest showed mediastinal vascular calcification and minimal left pleural thickening. There was also scattered alveolar pattern, an atelectatic band in the left posterior basal segment, and rounded atelectasis. Additionally, a 0.9 mm pleural-based mass lesion with a narrow neck, likely due to a past lung infection, was noted.

### Rheumatology consultation and management

The patient was referred to the rheumatology department for further clinical assessment. After a full assessment in the rheumatology clinic, he was first diagnosed with limited ANCA-related vasculitis based on the presence of refractory panuveitis.

A definitive diagnosis of GPA could not be established due to the lack of other systemic manifestations. And on the 23rd of June 2022 He was started on induction prednisolone therapy of 60 mg daily, alongside methotrexate 20 mg weekly with folic acid.

His second visit, fourty days later on the 2nd of august 2022, after his reassessment; revealed the persistence of symptoms and uveitic activity in the form of 1 + anterior chamber reaction and vitreous haze. At this point, the rheumatologist and the ophthalmologist agreed to start rituximab for the refractory ocular and neurological manifestations. Four weeks after receiving the two induction doses of rituximab, the patient returned for reassessment on the 12th of December 2022 and still exhibited active inflammation in both eyes.

A diagnosis of refractory c-ANCA-related vasculitis was made, and cyclophosphamide was recommended as second-line therapy.

**Fig. 3:**
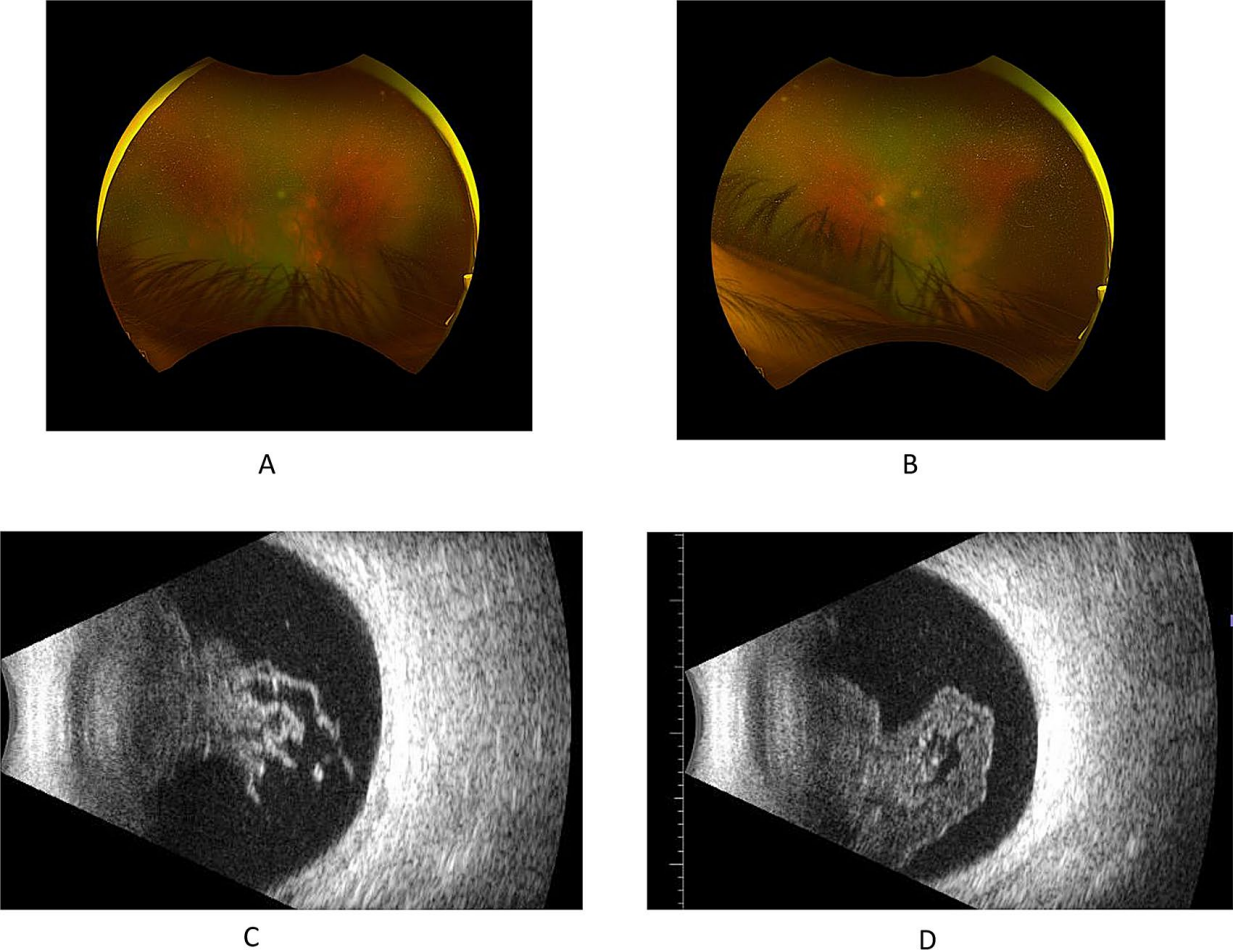
Visit Date 7/8/2023. Optos fundus photos 3 months after starting cyclophosphamide 10 mg. (**A**) Right eye showing active uveitis with increasing vitreous opacity and further drop in vision. (**B**) Left eye showing active uveitis with increasing vitreous opacity and further drop in vision. (**C**) B-Scan of the right eye and (**D**) left eye showeing dens vitrous opacity with mild subvitreal opacity (D)

**Fig. 4:**
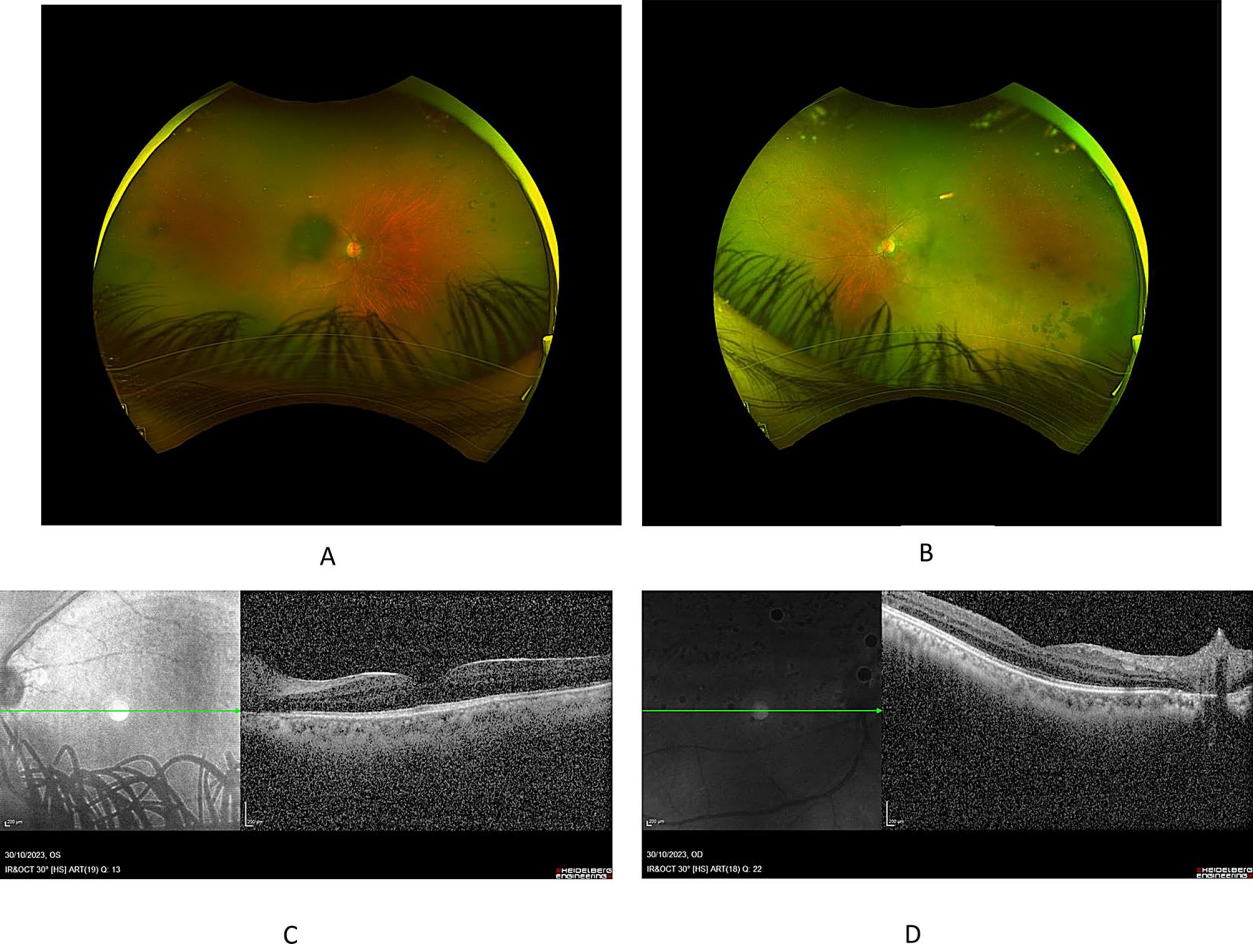
Visit Date: 30/10/2023. Optos fundus photos after receiving six doses of cyclophosphamide. (**A**) Right eye. (**B**) Left eye. The uveitis activity has begun to show a response to treatment with a better view of the posterior pole despite longstanding vitreous opacities. (**C**) Right eye (**D**) left eye optical coherence tomography showed resolved subretinal fluid with no cystoid macular edema

Upon re-evaluation in the uveitis clinic, three months after starting cyclophosphamide, on the 7th of August 2023 the patient failed to respond with a further drop in his vision. Visual acuities have been dropped to 20/400 in the right eye and 6/200 in the left eye. Biomicroscopic examination revealed increasing anterior chamber inflammation and denser vitreous opacities obscuring fundus assessment (Fig. 3A, B). B-Scan was performed, and it showed dens vitreous opacity with mild sub-vitreous opacity but no retinal or choroidal detachment (Fig. 3C, D). A decision was made to increase the prednisolone dose again to 60 mg daily. Two months later, on the 30th of October 2023, the patient had received six doses of cyclophosphamide and had begun to show clinical improvement. The patient had no pain or redness during this last visit and reported better vision. Clinical examination showed 20/80 and 20/50 visual acuities in the right and left eye, respectively. Biomicroscopy revealed only trace mixed cells in both eyes without flare. With no vitreous haze and a better view to the fundus despite the longstanding vitreous opacities. OCT showed resolved subretinal fluid with no cystoid macular edema (Fig. 4A, B, C, D).

## Discussion

In the present case, we report a middle-aged male who presented with persistent attacks of panuveitis over years and recurrent minor strokes with a strongly positive c-ANCA titer. Despite the absence of typical respiratory and renal system involvement of GPA and the lack of a biopsy, the final diagnosis was c-ANCA-related panuveitis. This aligns with a study that identified c-ANCA in uveitic cases where patients did not show signs of GPA [7]. That study found c-ANCA most prevalent in intermediate uveitis, followed by anterior uveitis, posterior uveitis, and panuveitis at 1%, highlighting the rarity of the condition presented in our case [7].

Among AAV, inflammatory ocular disease was more frequently the initial manifestation in MPO-ANCA-associated disease (69%) and negative-ANCA disease (67%) compared to PR3-ANCA-associated disease (45%) [2].

In their study, Watkins et al. identified uveitis in 17.9% of patients with AAV. Half of these cases also presented with scleritis, suggesting that the uveitic component commonly arises as a secondary complication [4].

In line with our report, a case of granulomatous panuveitis with retinal vasculitis and cystoid macular edema was documented in the literature as the initial manifestation of GPA, however it responded well to treatment with mycophenolate mofetil [8]. Another report in the literature described occlusive retinal vasculitis as the sole presenting sign of ocular GPA, which was also treated successfully with azathioprine and rituximab [9]. In contrast, our case presented with persistent bilateral anterior uveitis and dense vitreous opacities, in addition to mild macular edema along with peripheral chorioretinal scars. Despite treatment with methotrexate and rituximab, the patient showed inadequate response and only moderate improvement after six cycles of cyclophosphamide. At the time of reporting this case, the patient is still steroid-dependent. This raises a concern whether c-ANCA contributes to treatment resistance.

Ocular manifestations in patients with positive c-ANCA span a spectrum, including mild cotton-wool spots, intraretinal hemorrhages, necrotizing retinal vasculitis [10], and occlusion of the retinal artery or vein [11–14]. Although anterior segment manifestations, such as conjunctivitis, scleritis, iritis, and peripheral ulcerative keratitis (PUK), are more common and varied in these patients than posterior segment involvement [10, 14], with scleritis having the highest association with positive c-ANCA [15]. Our patient did not show any scleral or corneal involvement apart from chronic uveitic activity and its sequelae.

Interestingly, our patient had a history of repeated ischemic strokes and was on maintenance aspirin and rivaroxaban. Xiaoyan Li and colleagues identified a significantly elevated incidence of recurrent ischemic stroke in ANCA-positive patients. This heightened recurrence is likely attributable to persistent alterations in blood coagulation and endothelial damage within cerebral vessels. Their study revealed that out of 131 ANCA-positive patients, 93 individuals, accounting for 70.99%, experienced recurrent ischemic strokes [16]. 

Neurological involvement in vasculitis can vary significantly based on the specific type affecting the central nervous system (CNS), peripheral nervous system (PNS), and cranial nerves. For CNS involvement, the prevalence ranges from 5 to 15% of patients. In contrast, PNS involvement is more common, affecting 60–70% of patients, particularly those with Eosinophilic Granulomatosis with Polyangiitis (EGPA) and 15–50% of patients with GPA. Cranial nerve involvement is notably frequent in GPA, occurring in up to 15% of patients [17]. 

Autoimmune hemolytic anemia (AIHA) in the context of ANCA-related vasculitis is an uncommon occurrence, with limited evidence connecting the two conditions. The primary hematological manifestation associated with c-ANCA-related vasculitis is microangiopathic hemolytic anemia (MAHA), characterized by hemolysis resulting from microvascular injury. However, MAHA is rare in these patients [18]. 

Therefore, the combination of clinical features in this patient—ischemic strokes, autoimmune hemolytic anemia (AIHA), and panuveitis—represents a potentially novel association that warrants further investigation. This unique constellation of symptoms suggests additional studies to explore and clarify any underlying connection.

## Conclusion

In conclusion, this case report highlights an atypical presentation and clinical course of c-ANCA-related panuveitis. The presence of both ocular and neurological manifestations may indicate a more aggressive disease course, requiring urgent and intensive immunosuppressive therapy to control the inflammation and prevent further complications.

## Data Availability

No datasets were generated or analysed during the current study.
